# Adjustment of costly extra-group paternity according to inbreeding risk in a cooperative mammal

**DOI:** 10.1093/beheco/arv095

**Published:** 2015-07-03

**Authors:** Hazel J. Nichols, Michael A. Cant, Jennifer L. Sanderson

**Affiliations:** ^a^School of Natural Science and Psychology, Liverpool John Moores University, Liverpool L3 3AF, UK and; ^b^College of Life and Environmental Sciences, University of Exeter, Penryn TR10 9FE, UK

**Keywords:** extra-group paternity, extrapair paternity, intergroup interaction, mammal, mating system, warfare.

## Abstract

Females of many animal species seek mating opportunities with multiple males, despite being able to obtain sufficient sperm to father their offspring from a single male. In animals that live in stable social groups, females often choose to mate outside their group resulting in extra-group paternity (EGP). One reason proposed to explain female choice for extra-group males is to obtain compatible genes, for example, in order to avoid inbreeding depression in offspring. The benefits of such extra-group paternities could be substantial if they result in fitter, outbred offspring. However, avoiding inbreeding in this way could be costly for females, for example, through retaliation by cuckolded males or through receiving aggression while prospecting for extra-group mating opportunities. We investigate the costs and benefits of EGP in the banded mongoose *Mungos mungo*, a cooperatively breeding mammal in which within-group mates are sometimes close relatives. We find that pups born to females that mate with extra-group males are more genetically heterozygous are heavier and are more likely to survive to independence than pups born to females that mate within their group. However, extra-group matings also involve substantial costs as they occur during violent encounters that sometimes result in injury and death. This appears to lead femalebanded mongooses to adaptively adjust EGP levels according to the current risk of inbreeding associated with mating within the group. For group-living animals, the costs of intergroup interactions may help to explain variation in both inbreeding rates and EGP within and between species.

## Lay Summary

Female-banded mongooses risk their lives to mate with rivals during pack “warfare.” Data from wild banded mongooses reveal that 18% of pups are fathered by males from rival packs. These pups are less likely to be inbred are heavier and have higher survival chances than their within-pack counterparts. However, their mothers risk a lot to mate with extra-pack males; aggressive encounters between packs account for 20% of pup deaths and 12% of adult deaths.

## INTRODUCTION

Females often choose to mate with multiple males despite being able to obtain sufficient sperm to fertilize their eggs from a single male. Why they do so is not immediately obvious and consequently has been a topic of much debate (Akçay and Roughgarden 2007; Forstmeier et al. 2014). Among animals that live in stable groups, females often copulate with males outside their social unit (Griffith et al. 2002). Among birds, most of which are socially monogamous (Cockburn 2006), extra-group paternity (EGP) is known as extrapair paternity (EGP when the group size is 2), whereas among mammals, individuals tend to live in groups, so the term EGP is generally applied (Isvaran and Clutton-Brock 2007).

Females can benefit from seeking multiple mates in 2 main ways. First, females may obtain direct benefits from mating multiply. For example, the paternity uncertainty created through polyandrous mating can lead to an increase paternal care (Goldizen 1987; Santos and Nakagawa 2013) or a reduction in the probability of infanticide (Lukas and Huchard 2014). Second, females may gain genetic benefits for their offspring through obtaining “good genes” or “compatible genes” from a male other than her social partner or the dominant male in her territory (Foerster et al. 2003). Good genes are those that may be associated with heritable traits related to male attractiveness, survivability, or competitive ability (Forstmeier et al. 2014). If females are mating for good genes, they may either select a mate with particularly advantageous traits, or may mate multiply as a form of genetic bet-hedging (Fox and Rauter 2003; Forstmeier et al. 2014). Compatible genes are often thought to be those that lead to genetically heterozygous offspring, because heterozygosity reduces the likelihood of suffering from inbreeding depression (Hoffman et al. 2007). Females seeking compatibility should therefore attempt to mate with males that are genetically dissimilar to themselves. In accordance with this hypothesis, many studies have revealed that extrapair or group mates are less related to females than their within-pair mates (Blomqvist et al. 2002; Foerster et al. 2003; Brouwer et al. 2011; Arct et al. 2015), although not in every case; (Harrison et al. 2013; Hsu et al. 2015). It is also important to note that outbreeding depression is possible where strong local adaptation is present, hence females may not always be selected to maximize offspring heterozygosity. However, this appears to be relatively rare (Frankham et al. 2011).

The benefit of seeking compatible genes may be particularly important in species where potential mates are close relatives. In many cooperatively breeding species, high levels of natal philopatry mean that potential mates from within the group are often closely related (Koenig and Haydock 2004). Here, EGP can be an important mechanism of inbreeding avoidance. For example, in splendid fairy-wrens *Malurus splendens* and superb fairy-wrens *Malurus cyaneus*, many social pairs are first order relatives. In these species, inbreeding is avoided through an exceptionally high rate of EGP (more than 70%) (Koenig and Haydock 2004). Similarly, in pilot whales *Globicephala melas* and killer whales *Orcinus orca*, both sexes are philopatric, leading to high within-group relatedness. Here, all mating appears to be extra-group (Amos et al. 1991; Pilot et al. 2010).

Although polyandrous mating can benefit a female, mating with males other than their social partner or the resident dominant male may come at a cost. In some species, high predation levels lead to constraints on prospecting for mates (Bennett and Faulkes 2000). Studies have also shown that females who mate extra-group can have reduced paternal care for their offspring (Suter et al. 2009; García-Navas et al. 2013), or increased aggression from their social partner (McKibbin et al. 2011; García-Navas et al. 2013; Hoi et al. 2013). Females may also risk losing offspring if they are left unattended while seeking an extra-group mate (Hoffman et al. 2007). In species where territory borders are defended aggressively, attempts to encounter other social groups can be particularly risky (Watts et al. 2006). If an aggressive interaction occurs, females or their offspring may be injured or killed and, even if receptive females are not targeted, the death of other group-members will lead to a reduction in group size which can in-turn impact on territory size and survival (Kokko et al. 2001).

Although the costs of EGP may be an important determinant of whether or not females seek extra-group matings, this possibility has received little attention (Forstmeier et al. 2014). Here, we investigate the costs and benefits of EGP in a social mammal; the banded mongoose *Mungos mungo*. This species lives in large mixed sex groups of 5–40 adults (mean group size = 29) and has a polygynandrous mating system, with each group containing a “core” of 1–5 dominant breeders of each sex, along with younger subordinates that breed occasionally (Cant et al. 2013). New groups are formed when a cohort of males from 1 natal group joins a cohort of females from another natal group; hence, group-founders are closely related within each sex but unrelated between the sexes (Nichols et al. 2012). Although both males and females sometimes disperse from their natal groups, both sexes often remain philopatric. This, along with the death of group-founders, leads to a build-up of relatives in the group over time since the group was founded (Nichols et al. 2012). By the time, a group reaches 10 years old, the mean level of genetic relatedness between opposite-sex adult group-members is 0.25 (Nichols et al. 2012). Consequently, females that breed within their natal group often mate with relatives including fathers and brothers (Nichols et al. 2014). EGP could therefore be an important way in which inbreeding can be avoided in this species.

Banded mongoose groups generally breed 3–4 times per year (Cant et al. 2013). Female group-members enter estrus together (within 7 days of each other) and each female is guarded by a within-group male (Nichols et al. 2010). However, females are capable of refusing mating attempts and it does not appear to be possible for males to force female to mate (Cant 2000). Females are often able to escape their mate-guard to mate with other group-members (Cant 2000). Extra-group mating has been observed during intergroup encounters (Cant et al. 2002) but such mating is difficult to observe as it is often surreptitious and occurs in dense bushes. Nevertheless, EGP does occur in our study population, with extra-group males fathering 20% of pups (Nichols et al. 2014). A previous study (Cant et al. 2002) found that although 65% of intergroup encounters occur in areas of overlap between territories, both sexes are involved in initiating encounters by leading their group deep into neighboring territories: estrus females initiated 11% of intergroup encounters, whereas a further 24% were initiated by males (Cant et al. 2002). This leads to a higher intergroup encounter rate during estrus (Cant et al. 2002). During intergroup encounters, both resident and intruding females have been observed to mate extra-group (Cant et al. 2002). However, intergroup encounters are often violent and lead to injury and death, and may pose a risk to females or their offspring (Cant et al. 2002).

A previous study (Nichols et al. 2014) demonstrated that inbreeding is relatively common in the banded mongoose, with 14.3% of pups being moderately inbred (*F* = 0.125) and 8% of pups being highly inbred (i.e., the product of father–daughter and full-sibling matings, *F* = 0.25). Inbreeding appears to be influenced by female dispersal and mating patterns; the majority of pups (63.9%) are born to females breeding within their natal group, and these females often conceive to relatives, whereas females that mate-extra-group or disperse mate with nonrelatives (Nichols et al. 2014). The study also found that a significantly larger proportion of pups were fathered by extra-group males when females bred within their natal group in comparison to females that dispersed (Nichols et al. 2014). This highlights EGP as a potentially important means by which females could reduce their probability of inbreeding.

Here, we extend this work by investigating the costs and benefits of extra-group mating for female banded mongooses. Specifically, we test 1) whether pups fathered by extra-group males are genetically more heterozygous or more competitive than pups fathered by males within the group; 2) whether engaging in or seeking extra-group copulations involves costs to females; 3) whether females are more likely to seek EGP when the risk of inbreeding within groups is high.

## METHODS

### Study site and life-history data collection

Data were collected from a population of wild banded mongooses in Queen Elizabeth National Park, Uganda (0°12′S, 27°54′E) between 1997 and 2011. The climate is equatorial with little seasonal variation in temperature and 2 rainy seasons per year. Further details of habitat and climate are given elsewhere (Cant et al. 2013). All individuals in the study population were habituated to the presence of human observers at 2–4 m. Groups were visited every 1–4 days to collect behavioral and life-history data and are typically visited every day during oetrus, when intergroup interactions (IGIs) are most frequent. At each visit (lasting a minimum of 20min), the composition of the group was recorded. Life-history information, such as births, deaths, and dispersal events were recorded, and we knew accurate ages for the majority of the population. It was possible to distinguish death from dispersal as most dispersal events are induced through intense aggression from dominant group members (known as eviction) (Cant et al. 2001). Also, individuals disperse in single-sex cohorts and have never been observed to disperse alone, so the disappearance of a single individual with no prior signs of aggression was likely to be due to death (Cant et al. 2001). Where known or heavily implied, we recorded the cause of death.

Encounters between neighboring groups (IGIs) were recorded ad libitum. Intergroup encounters are described in detail elsewhere (Cant et al. 2002). In brief, when packs sight each other, they respond by standing erect and giving a distinctive, screeching call which alerts the rest of their pack to the presence of another group. When there are large size differences between the packs, the smaller group often flees. However, when groups are closely matched in size, individuals bunch together and approach the opposing group. Once groups are 20–30 m apart, they rush forward and engage in fights and chases. Fights are highly aggressive, involving biting and scratching, often to the head and legs. Attacks occur within and between the sexes (i.e., are not purely intrasexual). Occasionally, successful mating attempts have been observed to occur during these encounters. A video example of an IGI, including both fighting and mating is included in Supplementary Material (SI1).

One or two individuals in each group were fitted with a radio collar (Sirtrack Ltd, New Zealand). Individuals could be identified in the field by either color coded plastic collars or through unique patterns shaved or dyed in their fur on their backs. Shavings, collars, and dye patterns were maintained through regular trapping (every 3–6 months). Individuals were trapped using baited cage traps, and were anaesthetized using isoflurane or using intramuscular injections of 1mg/kg of ketamine and 0.8mg/kg of medetomidine, followed by an injection of 0.8mg/kg of atapamezol after handling (further details are given elsewhere: Hodge 2007, Jordan et al. 2010). Pups were first trapped at age 30–50 days. On first capture, permanent identification was made possible using either a uniquely coded tattoo or a pit tag, and a ~2-mm tail tip skin sample was collected for genetic analysis (Nichols et al. 2010). This trapping protocol was used more than 6000 times during the course of study without any individuals dying or becoming noticeably sick. This research was carried out under license from the Uganda National Council for Science and Technology and all procedures were approved by the Uganda Wildlife Authority.

### Genetic analysis

DNA was extracted from 1534 tail-tips by lysis with ProteinaseK, followed by phenol-chloroform purification (Sambrook et al. 1989) or using DNA extraction kits (Qiagen® Tissue and Blood Kit). Samples were genotyped at up to 20-microsatellite loci, isolated from a variety of carnivore species, including the banded mongoose (Supplementary Table SI2). Genotyping was conducted following (Nichols et al. 2010) or (post-2010) using multiplex PCRs (Qiagen® Multiplex PCR Kit, UK) with fluorescent-labelled forward primers and were visualized through fragment size analysis on an ABI 3730 DNA Analyzer. PCR conditions followed the Qiagen® Multiplex PCR Kit recommendations (but were conducted in 12-µL reactions), with an annealing temperature of 57 °C.

Values of pairwise relatedness were calculated following Lynch and Ritland (1999), and heterozygosity was calculated using HL following Aparicio et al. (2006). Parentage analysis was conducted using Cervus, version 3.0 (Marshall et al. 1998). As maternity could be narrowed down to a small number of females (mean = 4.3 per pup), maternities were assigned first. Several female group-members often gave birth in synchrony, and the subsequent litter is raised communally (Cant et al. 2013). As a consequence, all visibly pregnant females present in the group at the time of litter birth were included as candidate mothers to all pups born in the communal litter. For individuals where maternity was assigned at ≥95% confidence, paternity was then assigned assuming the maternity to be correct. All males in the study population more than 1-year old at litter conception (60 days before birth) were included as candidate fathers (mean = 72.5 per pup). In order to establish the confidence level of each assignment, Cervus conducts simulations of parentage assignment. Simulations took into account the relatedness structure of the banded mongoose population, with all candidate mothers being related to the real mother by 0.25, and 10% of candidate fathers being related to the real father by 0.2. Of the 1131 pups included in parentage analysis, maternities were assigned to 906 pups at ≥95% confidence and paternities were assigned to 629 of these pups at ≥95% confidence (equivalent to ≥90% confidence after taking into account the probability of mis-assigning the maternity).

### Statistical analyses

All statistical analyses were carried out using R 3.0.1 using either the lme4 or glmmADMB packages (Fournier et al. 2011; Bates et al. 2013). General linear mixed effect models (LMMs) and generalized linear mixed effect models (GLMMs) were used to control for repeated measures within years, social groups, breeding attempts, and individuals (where appropriate). Response variables followed normal, binomial, or Poisson distributions and were fitted in models with identity, logit, and log link functions, respectively. When data were zero-inflated, models were fitted using the glmmADMB package (Fournier et al. 2011) and model comparisons were made using likelihood ratio tests. Full models containing all possible explanatory variables were constructed and were simplified by stepwise model simplification; variables with the lowest explanatory power were sequentially dropped from the model until only those variables explaining significant variation (*P* < 0.05) remained. All dropped variables were then put back into the minimal model 1 at a time to determine their level of nonsignificance. As some data (such as body weight) are only available from a subset of individuals, models varied in their sample sizes. In each model, we used the maximum sample size available to us. Details of the models fitted, including sample sizes are included in Tables 1–4, 6, and 7.

**Table 1:** A LMM investigating whether extra-group males produce less homozygous pups than within-group males

Factors affecting offspring homozygosity			
Model term	Average effect ± SE	Wald statistic (χ^2^)	*P*
EGP	−0.031±0.013	5.69	0.017
Constant	0.50±0.0078		

Random effects: group, litter, mother’s ID, father’s ID and year. *N* = 629 pups from 196 communal litters in 16 groups over 15 years, produced by 126 mothers and 138 fathers.

Pup homozygosity was fitted as a normally distributed response variable with EGP as an explanatory factor.

**Table 2:** LMMs investigating whether extra-group pups are heavier at emergence from the natal den (at 30–40 days old) and as yearlings (350–380 days old) than within-group pups

Model term	Factors affecting mean weight at emergence (aged 30–40 days)			Factors affecting mean weight as yearling (aged 350–380 days)		
	Average effect ± SE	Wald statistic (χ^2^)	*P*	Average effect ± SE	Wald statistic (χ^2^)	*P*
EGP	**30.03±12.75**	**5.28**	**0.022**	53.87±32.69	2.53	0.11
Number of pups in litter	−0.63±1.87	0.089	0.77	0.55±4.61	0.014	0.91
Rainfall (mm)	4.23±3.91	1.10	0.29	**−26.56±8.01**	**9.67**	**0.0019**
Group size	−1.28±1.05	0.49	0.48	4.80±2.90	2.36	0.12
Mother’s age	−0.17±0.23	0.50	0.48	0.86±0.64	1.71	0.19
Constant	187.53±9.01			1258.11±55.33		

Random effects: pack, year, litter ID, mother’s ID, father’s ID. *n* = 104 pups from 45 communal litters over 11 years in 6 packs, with 42 fathers and 34 mothers. *n* = 121 yearlings from 64 communal litters over 12 years in 7 packs, with 62 fathers and 54 mothers.

Measurements of body mass (grams) were fitted as a normally distributed response variables and whether or not the pup was fathered by an extra-group male was fitted as the main explanatory variable of interest in both models. The following were controlled for by fitting them as further explanatory variables: the number of pups in the communal litter, the size of the social group (number of individuals more than 1 year of age at birth of the pup), rainfall (mean rainfall in mm in 30 days prior to birth) and the mother’s age at pup birth (months). The values in bold are significant at a minimum of *P* < 0.05.

**Table 3:** GLMMs investigating whether extra-group pups are more likely to survive to nutritional independence (90-days old) and 1 year than within-group pups

Model term	Factors affecting survival to nutritional independence (90 days)			Factors affecting survival to 1 year		
	Average effect ± SE	Wald statistic (χ^2^)	*P*	Average effect ± SE	Wald statistic (χ^2^)	*P*
EGP	**0.83±0.38**	**5.43**	**0.020**	0.09±0.49	0.05	0.82
Number of pups in litter	−0.022±0.056	0.15	0.69	−0.08±0.05	2.59	0.11
Rainfall (mm)	**0.30±0.12**	**7.79**	**0.0052**	0.16±0.11	2.08	0.15
Group size	−0.029±0.031	0.86	0.35	−0.01±0.02	0.14	0.71
Mother’s age	0.0054±0.0064	0.72	0.40	0.01±0.01	0.80	0.37
Constant	−0.30±0.38			0.64±0.25		

Random effects: pack, year, litter ID, mother’s ID, father’s ID. *n* = 479 pups from 153 communal litters in 12 packs over 13 years, with 121 fathers and 100 mothers. *n* = 272 pups from 120 communal litters in 12 packs over 13 years, with 95 fathers and 77 mothers.

Whether or not pups survived (1 = survived, 0 = did not survive) was fitted as a binomial response variable and whether or not the pup was fathered by an extra-group male was fitted as the main explanatory variable of interest in both models. The following were controlled for by fitting them as further explanatory variables: the number of pups in the communal litter, the size of the social group (number of individuals more than 1 year of age at birth of the pup), rainfall (mean rainfall in mm in 30 days prior to birth) and the mother’s age at pup birth (months).

**Table 4:** A GLMM investigating whether EGP is more likely to occur after inter-group encounters

Factors influencing the probability of EGP			
Model term	Average effect ± SE	Wald statistic (χ^2^)	P
**Intergroup encounter**	**0.84±0.39**	4.62	**0.032**
Constant	−1.14±0.36		

Random effects: pack and year. *n* = 183L, 15 packs, 12 years.

Whether or not EGP was observed in a communal litter was included as a binomial response variable, and whether or not an intergroup encounter was observed during the estrus period (60±5 days prior to birth of the communal litter) was included as an explanatory variable.

## RESULTS

### Are pups fathered by extra-group males more competitive than within-group pups?

**Figure 1:**
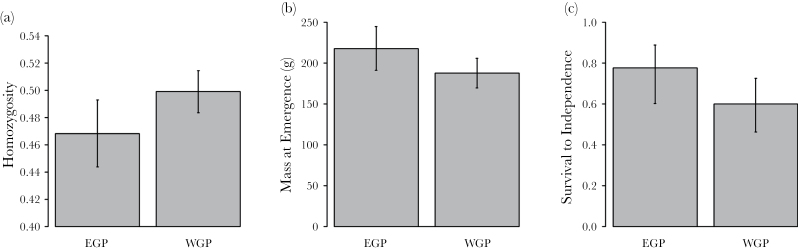
Effects of EGP on (a) offspring homozygosity, (b) offspring body mass at emergence (30–40 days), and (c) offspring survival to independence (90 days). Bars and confidence intervals show predicted means and standard errors, respectively (while controlling for a significant effect of rainfall on survival to emergence).

Parentage analysis uncovered 112 cases of EGP (17.8% of the 629 pups assigned a father). Pups that were the product of EGP were on average more genetically heterozygous than pups that are the product of within-group matings (LMM: $\chi_{\left(1\right)}^{2}$ = 5.69, *P* = 0.017, Table 1, Figure 1a). This is in accordance with previous work, which found that females mating with extra-group males were less related to their mates than females that mated within-group (Nichols et al. 2014).

Pups fathered by extra-group males were significantly heavier at emergence from the natal den (30–40 days old) than pups fathered by within-group males (LMM: $\chi_{\left(1\right)}^{2}$ = 5.28, *P* = 0.022, Table 2, Figure 1b). Furthermore, pups fathered by extra-group males were significantly more likely to survive to nutritional independence (90 days) than within-group pups (LMM: $\chi_{\left(1\right)}^{2}$ = 5.43, *P* = 0.020, Table 3, Figure 1c). However, there was no significant impact of EGP on weight as a yearling (LMM: $\chi_{\left(1\right)}^{2}$ = 2.53, *P* = 0.11, Table 2) or on survival to 1 year (LMM: $\chi_{\left(1\right)}^{2}$ = 0.05, *P* = 0.82, Table 3).

### Are there costs to females of extra-group mating?

**Figure 2:**
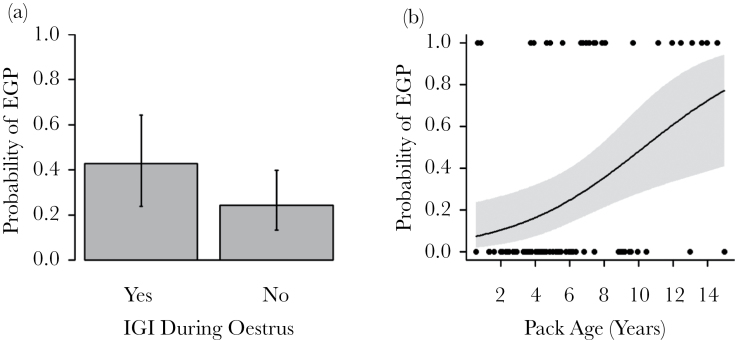
The effects of (a) an IGI occurring during group estrus and (b) pack age (years since the group was founded) on the probability of EGP occurring within a communal litter. Figures show predicted means and standard errors from 2 GLMMs.

Previous behavioral observations indicate that extra-group mating attempts primarily occur during aggressive encounters between neighboring groups (Cant et al. 2002). In accordance with this, we found that EGP was significantly more likely to be assigned in communal litters when an inter-group encounter was observed during the estrus period (LMM: $\chi_{\left(1\right)}^{2}$ = 4.62, *P* = 0.032, Table 4, Figure 2a).

To investigate the potential costs of engaging in IGIs, we quantified the proportion of individuals that were known to die due to IGIs. We found that, of the 687 individuals where cause of death is known (or heavily implied), a substantial proportion (15%) died during or following injury from intergroup encounters (Table 5). Pups (less than 90 days) appear to be particularly vulnerable during intergroup encounters; intergroup aggression accounts for 20% of pup deaths, compared with 12% of adult deaths, a significant difference (pups: 76/382, adults: 26/210, $\chi_{\left(1\right)}^{2}$ = 4.85, *P* = 0.028). However, there was no significant difference in the proportions of adult male and female (more than 1 year old) deaths in IGIs (males: 19/124, females: 7/86, $\chi_{\left(1\right)}^{2}$ = 1.80, *P* = 0.180). Together, this data suggest that females can suffer costs to engaging in intergroup encounters, including death, the loss of pups from previous litters and a reduction in group size which may in-turn impact on territory size and survival.

**Table 5:** Causes of death for 1808 banded mongooses, including 1103 pups (90 days and under) and 705 juveniles and adults

Cause of death	Number of individuals more than 90-days old	% of deaths with known cause	Number of pups (less than 90 days old)	% of deaths with known cause
IGI	30	10	76	20
Age/sickness/ generally weak	71	23	48	13
predated	155	51	200	52
Human induced	46	15	10	3
Eviction	2	<1	N/A	N/A
Giving birth	1	<1	N/A	N/A
Abandoned/ kidnapped	N/A	N/A	18	5
Within-group infanticide	N/A	N/A	30	8
unknown	400		721	
Total known	305		382	
Total	705		1103	

### Are females more likely to mate extra-group when the risk of inbreeding within groups is high?

Given the costs involved in extra-group mating, we predicted that females should seek mating opportunities outside their own group when there is a high risk of inbreeding by mating with within-group males. In support of this prediction, the probability of finding EGP in a communal litter was higher in older groups (LMM: $\chi_{\left(1\right)}^{2}$ = 9.57, *P* = 0.0020, Table 6, Figure 2b), which contain more relatives (Nichols et al. 2012). Once group-age had been taken into account, there was a nonsignificant trend for higher levels of EGP in groups with higher mean levels of relatedness between opposite sex adult group members (LMM: $\chi_{\left(1\right)}^{2}$ = 3.02, *P* = 0.082, Table 6).

**Table 6:** A GLMM investigating whether EGP is more likely to occur within a communal litter when the risk of inbreeding within a group is high (in older packs and when the mean relatedness between opposite-sex adults is high)

Factors affecting probability of EGP within litter			
Model term	Average effect ± SE	Wald statistic (χ^2^)	*P*
Number of (assigned) pups	**0.21±0.10**	**4.61**	**0.032**
Pack age (years)	**0.26±0.09**	**9.57**	**0.0020**
Mean male-female relatedness	8.36±4.87	3.02	0.082
Constant	−3.69 ±0.95		

Random effects: pack, year. *n* = 78 communal litters from 11 social groups over 12 years.

Whether or not EGP was detected in a litter was fitted as a binomial response variable (0 = no EGP, 1 = at least 1 extra-group pup). Pack age (years since the group was founded) and the mean level of relatedness between adult male and female group-members (aged at least 1 year) were fitted as explanatory variables. It may be particularly difficult to detect whether or not EGP has occurred in a litter when a small proportion of pups have been genotyped and/or assigned paternity. To reduce the probability of this affecting the results, this analysis was limited to litters where at least 50% of pups were genotyped and assigned paternity (78 out of possible 189 communal litters) and for the remaining litters, the number of assigned pups was included as an explanatory variable in the model. The values in bold are significant at a minimum of P < 0.05.

**Figure 3:**
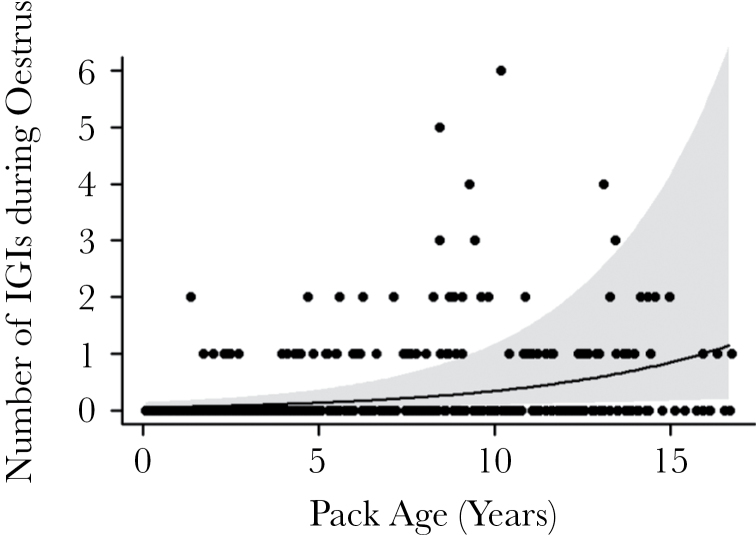
The impact of pack age (years since the group was founded) on the number of IGIs occurring during estrus (60±5 days before birth). Points show raw data whereas the line and shaded area show the predicted trend with confidence intervals from a GLMM while controlling for zero-inflation and the number of IGIs observed in a comparative time period after estrus (40±5 days before birth).

Early-life mortality resulting from inbreeding depression can potentially bias estimates EGP frequency (Reid et al. 2015). If offspring with extra-group fathers are less inbred and hence have higher survival chances than within-group offspring, mortality prior to genetic sampling could result in a spurious relationship between the probability of finding extra-group offspring and inbreeding risk. As we found evidence of lower early-life mortality in extra-group banded mongoose pups, it is likely that extra-group pups also have lower mortality prior to emergence from the den (and genetic sampling), making such biases likely in our system. The potential bias can be assessed by simulations, which take into account the probability of an offspring dying prior to genetic sampling (Reid et al. 2015). Unfortunately, in the banded mongoose, it is not possible to estimate the proportion of pups that die prior to sampling as females give birth in inaccessible underground dens and pups do not emerge for ~30 days, so litter-size at birth is unknown (Cant, et al., 2013). Instead, we sought to investigate whether females mate extra-group when they are at risk of inbreeding within groups is high using behavioral records of IGIs, which are not subject to biases in genetic sampling. We found that intergroup encounters were significantly more likely to occur during estrus in older groups (GLMM: $\chi_{\left(1\right)}^{2}$ = 13.66, *P* = 0.0002, Figure 3, Table 7) which contain more opposite-sex relatives (Nichols et al. 2012). However, there was no additional impact of average male–female relatedness on the numbers of IGIs that occur (GLMM: $\chi_{\left(1\right)}^{2}$ = 0.004, *P* = 0.95, Table 7).

**Table 7:** A GLMM investigating whether IGIs during pack estrus are more likely to occur within a communal litter when the risk of inbreeding within a group is high (in older packs and when the mean relatedness between opposite-sex adults is high)

Factors affecting number of IGIs during estrus			
Model term	Average effect ± SE	Deviance	*P*
IGI count outside of estrus	**0.24±0.09**	**6.54**	**0.011**
Pack age (years)	**0.16±0.05**	**13.66**	**0.0002**
Mean male–female relatedness	−0.11±1.52	0.004	0.95
Constant	−2.80±0.45		

Random effects: pack, year. *n* = 371 litters from 17 social groups over 17 years. The values in bold are significant at a minimum of P < 0.05. The number of IGIs during pack estrus was fitted as the response variable. Pack age (years since the group was founded) and the mean level of relatedness between adult male and female group-members (aged at least 1 year) were fitted as explanatory variables. The number of IGIs the group engaged in during a matched period outside of estrus was also included to control for background IGI rate.

## DISCUSSION

We found evidence of substantial benefits to females of mating with males from a different social group. First, pups fathered by extra-group males had higher levels of genetic heterozygosity than within-group pups. This is probably because extra-group mates are on average less related to the mother than within-group mates (Nichols et al. 2014), and hence extra-group pups are outbred in comparison to their within-group counterparts. Furthermore, we found that pups fathered by extra-group males are heavier at emergence from the den (30–40 days) than those fathered by within-group males. This early life weight advantage may have an important influence on survival because heavier pups are at an advantage when competing with their littermates for access to carers (Hodge et al., 2009). Accordingly, pups fathered by extra-group males were more likely to survive until nutritional independence (90 days) than pups fathered by within-group males. However, we did not find effects of EGP on weight and survival among yearlings, suggesting that the costs of inbreeding depression may be particularly high in early life. This result is in accordance with a study on the closely related meerkat, which found evidence for inbreeding depression on a range of early-life traits including pup mass at emergence and juvenile survival (Nielsen et al. 2012).

Although mating with extra-group males can be advantageous to banded mongoose females, these matings may come at a cost. Extra-group matings occurred during violent inter-group encounters, which account for a high proportion of adult and pup mortality (12% and 20% of known causes of death respectively, including females of breeding age). Females may therefore suffer costs to engaging in intergroup encounters including death, the loss of pups from previous litters and a reduction in group size which can in-turn impact on territory size and survival (Cant et al. 2002; Furrer et al. 2011). Furthermore, as banded mongooses breed regularly, females are pregnant for around 30% of each year (108±4.8 days per year, *N* = 199 females aged more than 1 year; Marshall H, unpublished data), so any injury is likely to have direct fitness consequences. Aggressive IGIs have been observed in other group-living carnivores and primates [Gray wolves *Canis lupus* (Cassidy 2013), Ethiopian wolves *Canis simensis* (Sillero-Zubiri and Macdonald 1998), African lions *Panthera leo* (Mosser and Packer 2009), spotted hyenas *Crocuta crocuta* (Boydston et al. 2001) common marmosets *Callithrix jacchus* (Lazaro-Perea 2001), chimpanzees *Pan troglodytes,* and humans *Homo sapien*s (Wrangham et al. 2006)]. In the majority of these species, aggressive interactions rarely involve matings, and instead appear to be related to intergroup competition over territory; killing or injuring rival group-members reduces the competitive ability of rival groups and hence increases the aggressors ability to acquire territory (Wrangham and Glowacki 2012). However, in a subset of these species, extra-group matings have been observed (common marmosets Lazaro-Perea 2001), or aggression toward opposite-sex intruders is rare [Ethiopian wolves (Sillero-Zubiri and Macdonald 1998), spotted hyenas (Boydston et al. 2001)], suggesting that individuals may use aggressive IGIs as an opportunity to prospect for mating opportunities. In the banded mongoose, territory gain is likely to be important in determining the frequency of aggressive IGIs (Cant et al. 2002; Furrer et al. 2011). However, the relationship between IGIs and EGP strongly suggests that access to mating opportunities is also important.

In species that have aggressive intergroup encounters, deaths are often biased toward adult males. For example, across 7 human subsistence farming societies, the median percentage of deaths due to intergroup warfare was 28.5% for males and 6.1% for females (Wrangham et al. 2006). Similarly, among chimpanzee societies, adult males are more than 6 times more likely to be the victims of lethal intergroup aggression than females (Wrangham et al. 2006). In contrast, for the banded mongoose, we found no significant differences between the proportion of adult males and females dying during intergroup encounters. This could be because intergroup encounters occur when entire groups meet, rather than on single-sex patrols as in chimpanzees (Wrangham and Glowacki 2012), hence females have little choice but to participate. Alternatively, these patterns may be due patterns of philopatry (Kitchen and Beehner 2007). In contrast to chimpanzees and humans (where females disperse) in banded mongooses both sexes can remain in their natal group for their entire lives and hence have high relatedness to the rest of their group (Nichols et al. 2012). Males and females may therefore gain equally from maintaining territory size and from reducing the group-size of rival groups.

In the banded mongoose, we found that the frequency of EGP increased with group age. This is consistent with the idea that estrus females may adaptively seek EGP when the probability of mating with a relative within the group is high (older groups contain more opposite-sex relatives Nichols et al. 2012). Higher levels of IGIs during estrus in older groups further support the idea that this relationship is due to variation in mating frequency, rather than being due to biases in early-life mortality (as suggested by Reid et al. 2015). Although group age had significant positive effect on the frequency of IGIs during estrus and on the probability of observing extra-group pups, mean male–female relatedness within the group did not. It is possible that group age is a better measure of inbreeding risk than mean relatedness as mean relatedness does not take within-group variance in relatedness into account, which could be important in governing mating decisions. Alternatively, banded mongooses may be unable to assess genetic relatedness directly, for example through scent cues (Mateo and Johnston 2000). Instead, they may use a simple rule governing when to mate extra-group, which is more closely associated with group age than it is to mean male–female relatedness. For example, female group founders may change their mating behavior over time as the number of related males (e.g., their sons and nephews) in the group increases. Natal females, on the other hand, may always assume that they are related to male group-members, and will mate extra-group where possible. Therefore, the proportion of females attempting to breed extra-group may increase over time since group formation due to an increase in the proportion of natal females, and changes in the behavior of group-founding females. Mechanisms of kin recognition will be the subject of future study. Although our results are consistent with adaptive female choice for nonrelatives, we cannot currently eliminate alternative explanations. For example, although females cannot be forced to mate (Cant 2000), they may be coerced into mating through threat of aggression during IGIs. This may explain why a small proportion of females mate extra-group even after dispersal from their natal group (Nichols et al. 2014). However, on average, females appear to benefit from extra-group matings through producing pups that are more genetically heterozygous, heavier and are more likely to survive until independence, suggesting that females may mate willingly with extra-group males.

Adaptive female mate-choice in order to receive compatible genes has been proposed in a number of vertebrate species, such as Antarctic fur seals *Arctocephalus gazella* (Hoffman et al. 2007) alpine marmots *Marmota marmota* (Cohas et al. 2008), European badgers *Meles meles* (Annavi et al. 2014), western sandpipers *Calidris mauri*, common sandpipers *Actitis hypoleuca* and Kentish plovers *Charadrius alexandrinus* (Blomqvist et al., 2002). Although there is strong evidence of adaptive mate choice for good or compatible genes in some cases, broader-scale patterns across birds, and mammals are not well supported (Griffith et al. 2002; Akçay and Roughgarden 2007; Hsu et al. 2015). For example, a meta-analysis by Akçay and Roughgarden (2007) found that fewer than half of studies supported adaptive extrapair paternity to gain good or compatible genes. This suggests that there may be additional factors influencing the distribution of EGP across species. For example, ecological or social constraints on mating opportunities may prevent females from mating extra-group and hence mask the effect of good or compatible genes (Akçay and Roughgarden 2007), or methodological differences between studies may impact on their ability to detect an effect (Arct et al. 2015). Alternatively, compatible genes may be particularly important in a subset of species, such as those where inbreeding is particularly likely to occur if females mate within their social system, as is the case in the banded mongoose.

## CONCLUSION

We show that female banded mongooses obtain genetic benefits from mating with extra-group males. Pups with extra-group fathers are more genetically heterozygous, heavier, and are have higher survival rates than pups produced by within-group males. However, extra-group mating comes at a cost. Intergroup encounters, where extra-group mating takes place, are highly aggressive and result in high levels of mortality, especially for pups. Females engaging in inter-group encounters therefore risk the loss of dependent pups, in addition to personal injury or death. As a consequence, females appear to strategically adapt their frequency of EGP according to current inbreeding risk, with EGP being more likely to be found in older social groups, which contain more relatives. Higher levels of IGIs during estrus in older groups support the idea that this relationship is due to variation in mating frequency, rather than simply on biases in early-life mortality. This study highlights the potential importance of the costs of EGP in determining the frequency of extra-group or pair paternity, which are rarely considered. The costs of obtaining extra-group mating partners may also contribute toward explaining variance in both inbreeding rates and EGP rates between species.

### Data accessibility

Microsatellite sequences are available from Genbank: accession numbers can be found in Supplementary Table SI2.

## SUPPLEMENTARY MATERIAL

Supplementary material can be found at http://www.beheco.oxfordjournals.org/

## FUNDING

This work was funded by a grant from the Natural Environment Research Council (NE/J010278/1).

## Supplementary Material

Supplementary Data
