# Supplementary material for: Adjustment of costly extra-group paternity according to inbreeding risk in a cooperative mammal
Source: Behav Ecol. 2015 Jul 3;26(6):1486–94. doi: 10.1093/beheco/arv095 (PMC4652740; doi:10.1093/beheco/arv095)
Supplement: Supplementary Data [file supp_26_6_1486__index.html]

Adjustment of costly extra-group paternity according to inbreeding risk in a cooperative mammal — Adjustment of costly extra-group paternity according to inbreeding risk in a cooperative mammal — Supplementary Data 

# Adjustment of costly extra-group paternity according to inbreeding risk in a cooperative mammal

## Supplementary Data

Data files

- Supplementary Data - Supplementary Data
- Supplementary Data - Supplementary Data
