# Supplementary material for: Adjustment of costly extra-group paternity according to inbreeding risk in a cooperative mammal
Source: Behav Ecol. 2015 Jul 3;26(6):1486–94. doi: 10.1093/beheco/arv095 (PMC4652740; doi:10.1093/beheco/arv095)
Supplement: Supplementary Data [file supp_arv095_Table_S1.docx]

Table S1. Details of the 20 microsatellites used in this study.

| **Locus** | **Species Isolated From** | **Number of Alleles** | **Polymorphic Information Content** | **Reference** | **Genbank Accession Number** |
| --- | --- | --- | --- | --- | --- |
| Ss11-12 | Meerkat (*Suricatasuricatta)* | 9 | 0.630 | Griffin et al. 2001 | AF271118 |
| Ss7-1 | Meerkat (*Suricatasuricatta)* | 5 | 0.613 | Griffin et al. 2001 | AF271115 |
| Ss10-4 | Meerkat (*Suricatasuricatta)* | 5 | 0.416 | Griffin et al. 2001 | AF271117 |
| Ss13-8 | Meerkat (*Suricatasuricatta)* | 7 | 0.376 | Griffin et al. 2001 | AF271120 |
| Mm5-1 | Banded mongoose (*Mungosmungo*) | 3 | 0.370 | Waldick et al. 2003 | AY142703 |
| Mm10-7 | Banded mongoose (*Mungosmungo*) | 3 | 0.368 | Waldick et al. 2003 | AY142693 |
| TGN | Banded mongoose (*Mungosmungo*) | 6 | 0.319 | Waldick et al. 2003 | AY142696 |
| A248 | Banded mongoose (*Mungosmungo*) | 4 | 0.255 | Waldick et al. 2003 | AY155580 |
| M53 | Banded mongoose (*Mungosmungo*) | 4 | 0.303 | Waldick et al. 2003 | AY142700 |
| A226 | Banded mongoose (*Mungosmungo*) | 2 | 0.182 | Waldick et al. 2003 | AY142694 |
| AHT130 | Domestic dog (*Canislupus familiaris*) | 3 | 0.503 | Griffin et al. 2001 | NA |
| Hj35 | small Asian mongoose (*Herpestesjavanicus*) | 10 | 0.687 | Thulin et al. 2002 | AY090498 |
| Ag6 | Antarctic fur seal (*Arctocephalusgazella*) | 5 | 0.463 | Hoffman et al. 2008 | EU045417 |
| Ag8 | Antarctic fur seal (*Arctocephalusgazella*) | 3 | 0.570 | Hoffman et al. 2008 | EU045419 |
| Agt25 (FS15) | Antarctic fur seal (*Arctocephalusgazella*) | 2 | 0.358 | Hoffman & Nichols 2011 | JF746980 |
| Agt44 (FS44) | Antarctic fur seal (*Arctocephalusgazella*) | 4 | 0.462 | Hoffman & Nichols 2011 | JF746986 |
| FS50 | Antarctic fur seal (*Arctocephalusgazella*) | 3 | 0.276 | Hoffman & Nichols 2011 | JF746991 |
| Hic1-95 | Egyptian mongoose (*Herpestes ichneumon)* | 5 | 0.643 | Rodrigues et al. 2009 | FJ357430 |
| Hic2-52 | Egyptian mongoose (*Herpestes ichneumon)* | 7 | 0.528 | Rodrigues et al. 2009 | FJ357432 |
| Hic4-30 | Egyptian mongoose (*Herpestes ichneumon)* | 8 | 0.379 | Rodrigues et al. 2009 | FJ357438 |
